# Clinical and Technical Overview of Preimplantation Genetic Diagnosis for Fragile X Syndrome: Experience at the University Hospital Virgen del Rocio in Spain

**DOI:** 10.1155/2015/965839

**Published:** 2015-12-02

**Authors:** Raquel M. Fernández, Ana Peciña, Maria Dolores Lozano-Arana, Beatriz Sánchez, Juan Carlos García-Lozano, Salud Borrego, Guillermo Antiñolo

**Affiliations:** ^1^Department of Genetics, Reproduction and Fetal Medicine, Institute of Biomedicine of Seville (IBIS), University Hospital Virgen del Rocío/CSIC/University of Seville, 41013 Seville, Spain; ^2^Centre for Biomedical Network Research on Rare Diseases (CIBERER), 41013 Seville, Spain

## Abstract

Fragile X syndrome (FXS) accounts for about one-half of cases of X-linked intellectual disability and is the most common monogenic cause of mental impairment. Reproductive options for the FXS carriers include preimplantation genetic diagnosis (PGD). However, this strategy is considered by some centers as wasteful owing to the high prevalence of premature ovarian failure in FXS carriers and the difficulties in genetic diagnosis of the embryos. Here we present the results of our PGD Program applied to FXS, at the Department of Genetics, Reproduction and Fetal Medicine of the University Hospital Virgen del Rocío in Seville. A total of 11 couples have participated in our PGD Program for FXS since 2010. Overall, 15 cycles were performed, providing a total of 43 embryos. The overall percentage of transfers per cycle was 46.67% and the live birth rate per cycle was 13.33%. As expected, these percentages are considerably lower than the ones obtained in PGD for other pathologies. Our program resulted in the birth of 3 unaffected babies of FXS for 2 of the 11 couples (18.2%) supporting that, despite the important drawbacks of PGD for FXS, efforts should be devoted in offering this reproductive option to the affected families.

## 1. Introduction

Fragile X syndrome (FXS, OMIM #300624) is the most common known monogenic cause of intellectual disability and affects all ethnic groups worldwide with an estimated frequency of about 1 in 4000–5000 [1, 2]. In >99% of cases, the disorder is caused by the unstable expansion of a CGG repeat in the 5′ untranslated region of* FMR1* gene, which leads to an abnormal hypermethylation, resulting in suppression of* FMR1* transcription and decreased protein levels in the brain [3, 4]. The boundaries between the* FMR1* allele categories are not precise and must be always made by considering both family history of each patient and repeat instability (Genereviews for* FMR1*-Related Disorders http://www.ncbi.nlm.nih.gov/books/NBK1384/). In general, normal* FMR1* alleles present a number of CGG repeats varying from 5 to 44, have no meiotic or mitotic instability, and are generally transmitted without any variation in repeat number. Borderline alleles present approximately 45–54 repeats and although they do not cause FXS in 14% of cases are unstable and may expand into the premutation range when transmitted by the mother [5]. Alleles of 55–200 repeats or premutation alleles are not associated with FXS either, but potential repeat instability upon their transmission may result in full-mutation alleles in the descendants (>200 repeats). In addition, these premutation alleles do convey increased risk for fragile X-associated tremor/ataxia syndrome (FXTAS), more frequently in males, and primary ovarian insufficiency (POI) in females. Finally, full-mutation alleles (200-several thousand repeats) are associated with aberrant hypermethylation of the* FMR1* promoter and are clearly associated with FXS. While the phenotype of males with an* FMR1* full mutation depends almost entirely on the number of repeats and subsequent degree of the promoter methylation, the phenotype of females with an* FMR1* full mutation also depends on the random X-chromosome inactivation. Therefore,* FMR1* full mutations almost always result in moderate to severe intellectual disability (ID) in males and mild ID to normal intellect in females. In addition, affected males may also have a characteristic facial appearance (long face, large ears, and prominent jaw), joint laxity, and macroorchidism. Behavioral abnormalities, sometimes including autism spectrum disorder, are also very common.

Reproductive options for the couples with familial history of FXS include prenatal diagnosis followed by possible termination of an affected pregnancy. However, the decision whether or not to terminate is frequently hard and difficult, especially since the prediction of phenotype is not possible because of mosaicism in males and X-inactivation ratios in females.

An alternative strategy for couples is preimplantation genetic diagnosis (PGD). PGD consists in the genetic analysis of at least one blastomere taken from* in vitro* fertilized embryos either on day 3 at the cleavage stage or at the blastocyst stage. Only unaffected embryos are transferred, avoiding the physical and psychic traumatism of the termination of pregnancies in the case of affected fetuses detected later by prenatal diagnosis. Most importantly, it avoids the difficult decision whether or not to terminate in the case that the fetus is female carrier, situation in which it is not possible to predict the final phenotype of the girl. Nevertheless, the PGD option has also important disadvantages and/or drawbacks, especially in the context of this disease, as it will be further discussed [6, 7]. Here we present the results of our program of PGD of FXS. All the procedures were performed at the University Hospital Virgen del Rocio in Sevilla, Spain (HUVR).

## 2. Materials and Methods

### 2.1. Inclusion of Couples in Our PGD Program for FXS

Since 2010, a total of 16 couples requested their inclusion in our PGD Program for the selection of embryos free of FXS. During the first consultation, the couples should provide a clear and accurate genetic test report, confirming the carrier status of a* FMR1* premutation or full mutation for the female. Extensive Genetic Counselling and information about the PGD procedures, success rate and possibility of misdiagnosis inherent to techniques, are then given by our multidisciplinary team of geneticists, embryologists, and gynaecologists to the couples. Informed consent concerning PGD and related procedures as well as the fate of the nontransferred embryos must be signed by the couples. Then, a basic test is prescribed to evaluate the reproductive state of the couples, which includes a hormone analysis and transvaginal ultrasound in the female, seminogram in the male, and serology for hepatitis B and C, HIV, and syphilis in both of them. Of the 16 initial couples, 5 were excluded of our program because of serum FSH levels >14 mUI/mL or estradiol levels >60 pg/mL and/or at least two consecutive positive results for the clomiphene citrate challenge test. The remaining 11 couples (9 with a* FMR1* premutation and 2 with a* FMR1* full mutation) had at least 1 PGD cycle.

PGD for FXS is carried out in our center by indirect molecular analysis using short tandem repeats (STRs) located within or in the neighbouring regions of the* FMR1* gene. Therefore, once the results of the basic test meet with the minimal quality requirements for the assisted reproduction techniques, DNA samples are extracted from peripheral blood of both members of the couple as well as of any other family member (usually an affected male) to proceed with the selection of informative genetic markers in the context of each family. Finally, when such selection has been achieved and the “disease haplotype” has been clearly identified warranting the reliable diagnosis of future embryos, the couple starts with the assisted reproduction protocol. Assisted Reproductive Techniques and embryo biopsy followed by our center have been previously described [8, 9].

### 2.2. Determination of the Number of FMR1 CGG Repeats in the Female Carriers

In order to determine the number of CGG repeats within the 5′UTR region of* FMR1* in the females of the couples, we used the AmplideX FMR1 PCR KIT (Asuragen Inc., Austin, USA) for the amplification of such region, following the manufacturers' recommendations. This kit has been designed to detect the full range of* FMR1* CGG expansions (including premutations and full-mutations) by PCR and capillary electrophoresis [10, 11].

### 2.3. Selection of Markers for Indirect Molecular Diagnosis of FXS and Multiplex PCR Protocol

A panel of seven polymorphic STRs located within the* FMR1 *gene or surrounding it was selected to perform indirect molecular analysis of FXS. These markers (*DXS998, DXS548, FRAXAC1*,* FRAXAC2, DXS1215, DXS6687, *and* DXS1193*) had been previously used for PGD for FXS [12, 13] (Supplementary Figures  1 and 2 in Supplementary Material available online at http://dx.doi.org/10.1155/2015/965839).

Primers for the amplification of those markers were designed using the Primer3 software (http://primer3.sourceforge.net/) so that they all could be simultaneously amplified with the same annealing temperature and conditions (primer sequences available on request).

A one-step multiplex single-cell fluorescent PCR is used for the simultaneous amplification of several combinations of the 7 markers, using the QIAGEN Multiplex PCR kit (QIAGEN, GmbH; Hilden, Germany) and an adaptation from a protocol previously described [14]. Optimal cell lysis protocol and PCR conditions were set up on single cells biopsied from supernumerary IVF embryos not suitable for transfer or cryopreservation. The optimized reaction mix contains 0,2 *μ*M of each primer, 5x Sol Q and 2x QIAGEN Multiplex PCR Master Mix, for a final volume of 25 *μ*L. The PCR Program is as follows: 15 minutes at 94°C, 10 cycles of 30 seconds at 96°C, 30 seconds at 61.5°C, and 30 seconds at 72°C, followed by 35 cycles of 30 seconds at 94°C, 30 seconds at 61.5°C, and 30 seconds at 72°C, and a final extension of 15 minutes at 60°C. PCR products are analyzed on an ABI3730 automated sequencer (Applied Biosystems, Foster City, CA).

### 2.4. Multiple Displacement Amplification (MDA) on Single Cells

We adapted the protocol described by Kumar et al. in 2008 [15], to obtain Whole Genome Amplification (WGA) of the blastomeres biopsied from the embryos resulting from the PGD-cycle of one premutation female carrier that requested sperm donation. The optimized adapted protocol for MDA has been previously described by our group [9].

### 2.5. Haplotyping of the Embryos

After 30 min at −80°C, cells are lysed by incubation in 200 mM NaOH, 50 mM DTT lysis buffer for 10 minutes at 65°C.

The corresponding genetic analysis of the embryos is subsequently performed using the previously selected combination of markers and the described one-step multiplex fluorescent PCR protocol at the single-cell level.

## 3. Results 

### 3.1. Overall Clinical Results

The overall clinical results for the PGD cycles for FXS are summarized in Table 1.

A total of 15 cycles were performed for 11 couples. Worth of note, 4 out of those 15 cycles (26.67%) resulted in no embryos. Specifically, 3 out of those 4 cycles were the first PGD cycles for 3 of the couples, and the remaining one was the third cycle for another couple. These poor results led the PGD team to make the decision of not offering the possibility of additional PGD cycles to those couples.

The fertilization rate, considering the correctly fertilized oocytes out of the total number of mature injected oocytes, was 63.2% (55 out of 87 oocytes). A total of 43 out of the 55 embryos were analyzed (78.2%), with a very variable number of embryos analyzed per cycle, ranging from 0 to 12. Finally, 40 out of the 43 analyzed embryos (93%) were reliably diagnosed.

**Figure 1:**
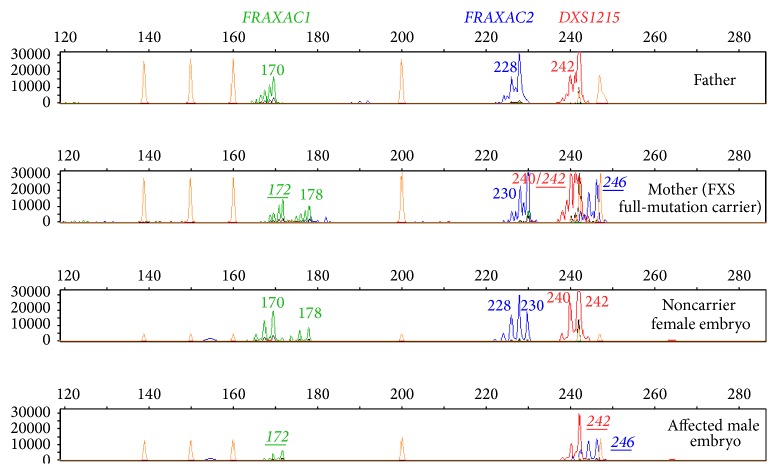
Electropherograms showing the profile of the STR-markers throughout the* FMR1* gene and its surrounding regions. Each lane shows the peaks obtained for each marker after the application of the specific multiplex fluorescent PCR method for FXS in a couple (lanes 1 and 2) and in the blastomeres biopsied from two of the embryos of their PGD-cycle (lanes 3 and 4). Each peak corresponds to the PCR product amplification of a marker, whose size depends on the specific number of repeats (the more the repeats the larger the PCR product). The sizes for each marker in each lane are also indicated with numbers. The sizes of the specific combination of alleles linked to the disease, which was identified by a previous informativity testing in the context of the family, are shown in italic and underlined characters.

Molecular analysis of the embryos was performed by fluorescent multiplex PCR of the STRs in 7 couples (9 cycles, 31 embryos) (Figure 1) and by a combination of MDA-PCR in a female carrier that requested sperm donation (1 cycle, 12 embryos). The decision of performing WGA on blastomeres biopsied from the embryos of this patient was taken due to the impossibility of* a priori *knowing if the STR alleles of the male donor were going to be informative enough to lead to a reliable diagnosis of the resulting embryos. Despite the inconveniences of an increased risk for contamination with external DNA sources and a considerably longer protocol, the availability of more DNA quantity derived from each blastomere obtained through MDA let us have the possibility of performing direct analysis of the CGG expansion if required. Nevertheless, no direct genotyping of the* FMR1* CGG expansion was necessary, since indirect analysis through the STRs was informative enough to determine the status of each embryo for the disease.

Finally, 12 embryos were transferred in 7 out of the 15 cycles, which correspond to a transfer rate of 46.67% (Table 1). Biochemical pregnancy (considered when *β*-hCG values ≥5 UI/L, 9 days after transfer) was achieved in 2 of the 15 cycles (13.33%), for 2 of the 11 couples (18.18%). Pregnancy was subsequently confirmed by echography in those 2 cases, leading to clinical pregnancy rate of 13.33% per initiated cycle and of 28.57% per transfer. Finally, birth at term of 3 unaffected children (all of them noncarrier females) for 2 couples was achieved, which corresponds to a live birth rate of 13.33% per initiated cycle and of 28.57% per transfer.

No supernumerary unaffected embryos were suitable to be cryopreserved.

### 3.2. Comparative Analyses of the Clinical Results

In general, when compared with a control group of 172 patients undergoing PGD cycles for other conditions, FXS carriers had considerably fewer metaphase II oocytes retrieved (median 5.8 MII oocytes retrieved for FXS* versus* median 10.5 MII oocytes retrieved for other diseases). This obviously leads to low transfer rates (46.67% for PGD-FXS* versus* 61.9% for PGD-other diseases) and low live birth rates per initiated cycle (13.33% for PGD-FXS versus 17.68% for PGD-other diseases).

On the other hand,* FMR1* CGG-repeat sizing had been performed for all the females, previous to their inclusion in our PGD Program for FXS. This procedure let us confirm their carrier status for either a premutation (9 women) or a full-mutation (2 women). While expanded alleles sizes within the range of full mutation were impossible to determine with the methodology employed, the alleles within the premutation range were successfully genotyped with a CGG repeats number varying from 55 to 104.

Small sample sizes did not let us reach statistical power to perform comparative analyses among both groups of patients (premutation carriers* versus* full-mutation carriers). Moreover, no association study could be performed between the number of CGG repeats and the number of oocytes retrieved. Nevertheless, it is important to note that the only two pregnancies achieved in this program corresponded to one of the two full-mutation carriers and to one premutation carrier with the number of CGG repeats in lower limit (55 repeats).

## 4. Discussion

We report our experience of using PGD for patients who carry a* FMR1* premutation or full mutation, summarizing data of 15 cycles in 11 women. One of our findings, comparing with PGD for other conditions, is that ovarian dysfunction in FXS carriers is a clear limitation leading to a high cancelation rate of embryo transfer and overall reduced efficiency of PGD for this disease, as previously reported [7]. Previous studies described that the risk for premature ovarian failure is higher in women having 59–99 CGG repeats, and thereafter the risk decreases for women with CGG repeats over 100 [7, 16, 17]. In fact, although our limited sample size has not let us perform a correlation study between the number of CGG repeats and ovarian response, our results are concordant with such findings: the three women with no resulting embryos from their first cycle presented 66, 75, and 85 CGG repeats, respectively. Moreover, the 2 women with the higher figures of good-quality biopsied embryos had 55 CGG repeats (12 embryos in one cycle) and >200 repeats (5 and 7 embryos in two consecutive cycles), respectively. Of note, both women resulted to be clinically pregnant and gave birth to unaffected children as a result of PGD cycles. When comparing our study with the largest detailed report on PGD for FXS published so far [7], the results are very satisfactory. In fact, our median values of MII oocytes obtained per oocyte retrieval (6.1 ± 4* versus* 7.4 ± 5) and our fertilization rate (63.2%* versus* 73%) were lower. However, the embryo transfer rates per started cycle were similar in both studies (46.67%* versus* 41%), and our clinical pregnancy rate per transfer (28.57%* versus* 17%) and live birth rate per transfer (28.57%* versus* 12%) were considerably higher. On the other hand, in 2012, the ESHRE PGD Consortium reported the results of 10 years of data collection from several international PGD centers [18]. In general, a total number of 4733 cycles for monogenic diseases were reported during their first 10 years of data collection leading to a clinical pregnancy rate of 29% per transfer, quite similar to the overall 28.57% achieved in our institution for PGD-FXS.

Regarding the method used for genetic analysis of the embryos, it also constitutes an additional difficulty for PGD-FXS. It has been widely demonstrated that pathogenic* FMR1* expanded alleles cannot be reliably amplified by standard PCR on a single cell, and therefore reliable direct analysis of the CGG repeats on the biopsied blastomeres could only be performed in those cases where normal alleles of the parents have different number of triplets [19]. In these circumstances, detection of only the paternal allele would be interpreted as an affected/carrier female, and absence of amplification would suggest an affected male, although allele dropout (ADO) cannot be ruled out. Unfortunately up to 40% of couples have been reported to be “noninformative” for direct analysis because both members of the couple carry similar CGG repeats, making the direct genetic analysis of the expansion unreliable [6]. For that reason, indirect analysis of multiple STRs linked to the disease in combination with the direct analysis of the triplets is an option chosen by some labs [13]. This option is useful to reduce the risk of misdiagnosis related to ADO and overcome the problem of uninformativity previously commented on but implies at least two rounds of PCR [6, 7, 13] or a WGA procedure previous to the specific PCR reactions [20], with the subsequent increased risk for contamination and a longer workup time. We decided to choose the best option minimizing the possibility of contamination with external DNA sources and time to obtain the results (around just 3 hours after the embryos biopsy) and optimized a one-step unique multiplex PCR for the simultaneous amplification of the informative STR markers for each couple. Predictive accuracy for indirect testing, avoiding misdetection of recombination events, is complete when intragenic or closely flanking markers are used, as it occurs with our method. The selection of informative markers for each couple, located within* FMR1* or flanking it on both its 5′ and 3′ regions, warranted the possibility of detecting any recombination event within the region and/or ADO events. However, none of such events were detected in the genetic analysis of any of the embryos. Just 3 of the 43 embryos could not be diagnosed due to eventual failures of either the cell lysis or the PCR procedures. Therefore, the % of accurately diagnosed embryos with the one-step multiplex PCR method was quite satisfactory. Nevertheless, we have also available the possibility of performing MDA for WGA as a previous step to the genotyping of the CGG repeats and the STRs for special cases as the previously commented. The disadvantage of the MDA approach is that it results more expensive and takes more than 1 day to obtain the results but warrants the availability of enough quantity of DNA to perform several genetic analyses (including the direct study of the CGG repeats expansion) if required.

## 5. Conclusions

Some centers have stopped offering PGD for FXS carriers or have suggested other alternatives such as egg donation, given the high prevalence of low response to hormonal stimulation in those females as well as the difficulties of the specific genetic test. The balance of our PGD Program applied to FXS since 2010, leading to the birth of unaffected babies for 2 of the 11 couples treated (18.18%), is quite satisfactory. Our success is in fact limited, since the two women that achieved clinical pregnancy in our program had little or no risk of fragile X primary ovarian insufficiency. Nevertheless, we can conclude that, despite the fact that PGD for FXS is particularly challenging, costs and efforts are worthwhile.

## Supplementary Material

Supplementary figures 1 and 2 show the specific location of the FMR1 gene and the STR markers used for PGD of FRAX.

## Figures and Tables

**Table 1:** Clinical data for PGD of FXS at HUVR.

	FXS
Number of couples treated	11
Maternal age	32.7 ± 3.4
Number of cycles performed	15
Number of cycles performed per couple	1.4 ± 0.7
Number of mature oocytes submitted to ICSI	87
Number of mature oocytes submitted to ICSI per cycle	6.1 ± 4.3
Number of oocytes fertilized	55
% of oocytes fertilized	63.22%
Number of oocytes fertilized per cycle	3.7 ± 3.7
Number of embryos analyzed	43
% of embryos analyzed	78.18%
Number of embryos analyzed per cycle	2.9 ± 3.4
Number of informative embryos	40
% of informative embryos	93.02%
Number of transfers	7
% of transfers	46.67%
Number of embryos transferred	12
Number of biochemical pregnancies	2
Number of clinical pregnancies	2
% of clinical pregnancies per cycle	13.33%
% of clinical pregnancies per transfer	28.57%
Implantation rate	25%
Number of pregnancies going to term	2
Number of babies born	3
Live birth rate per cycle	13.33%
Live birth rate per transfer	28.57%
