# Supplementary figures and images for: Clinical and Technical Overview of Preimplantation Genetic Diagnosis for Fragile X Syndrome: Experience at the University Hospital Virgen del Rocio in Spain
Source: Biomed Res Int. 2015 Dec 2;2015:965839. doi: 10.1155/2015/965839 (PMC4680048; doi:10.1155/2015/965839)

Supp. Figure 1

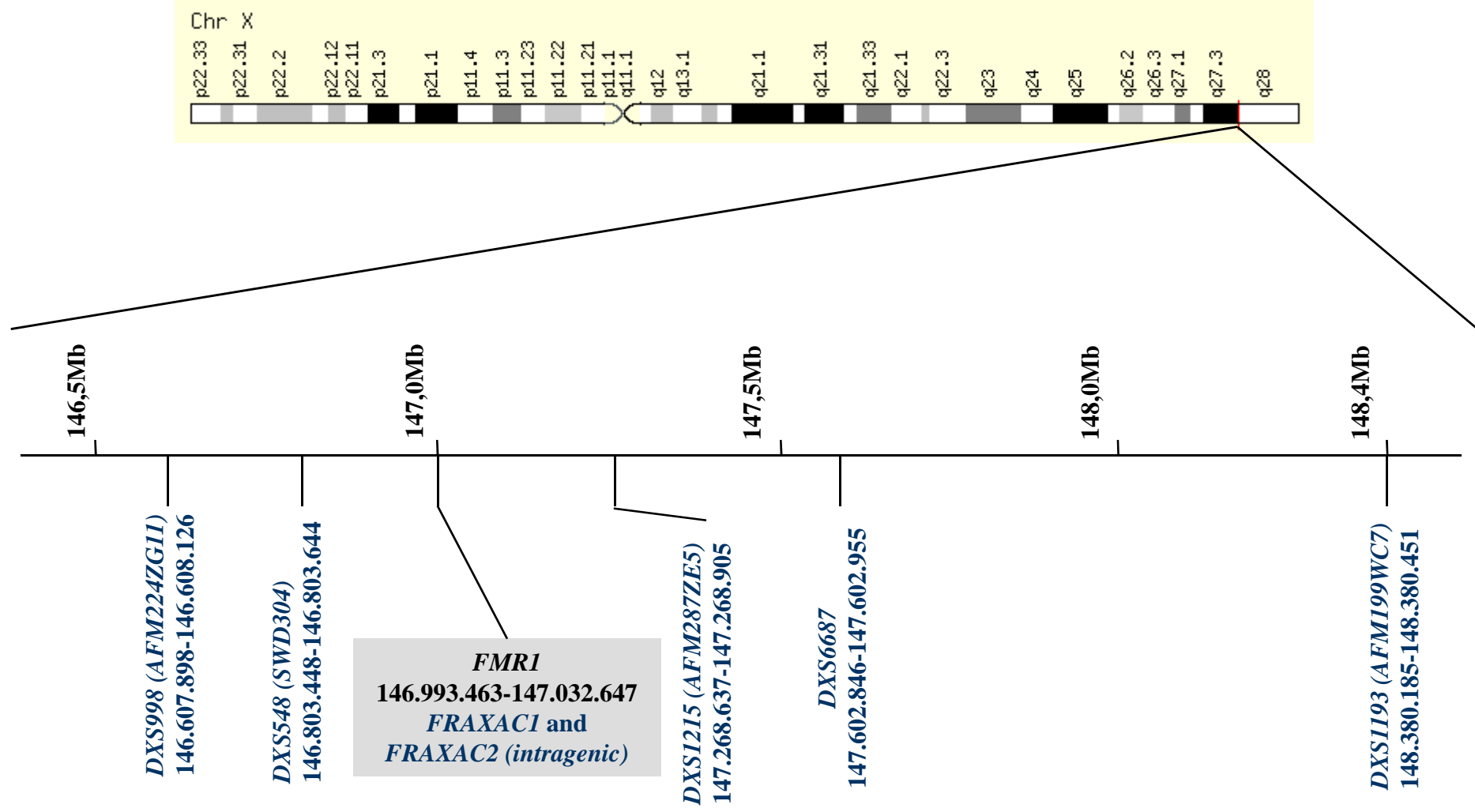

# Supp. Figure 2

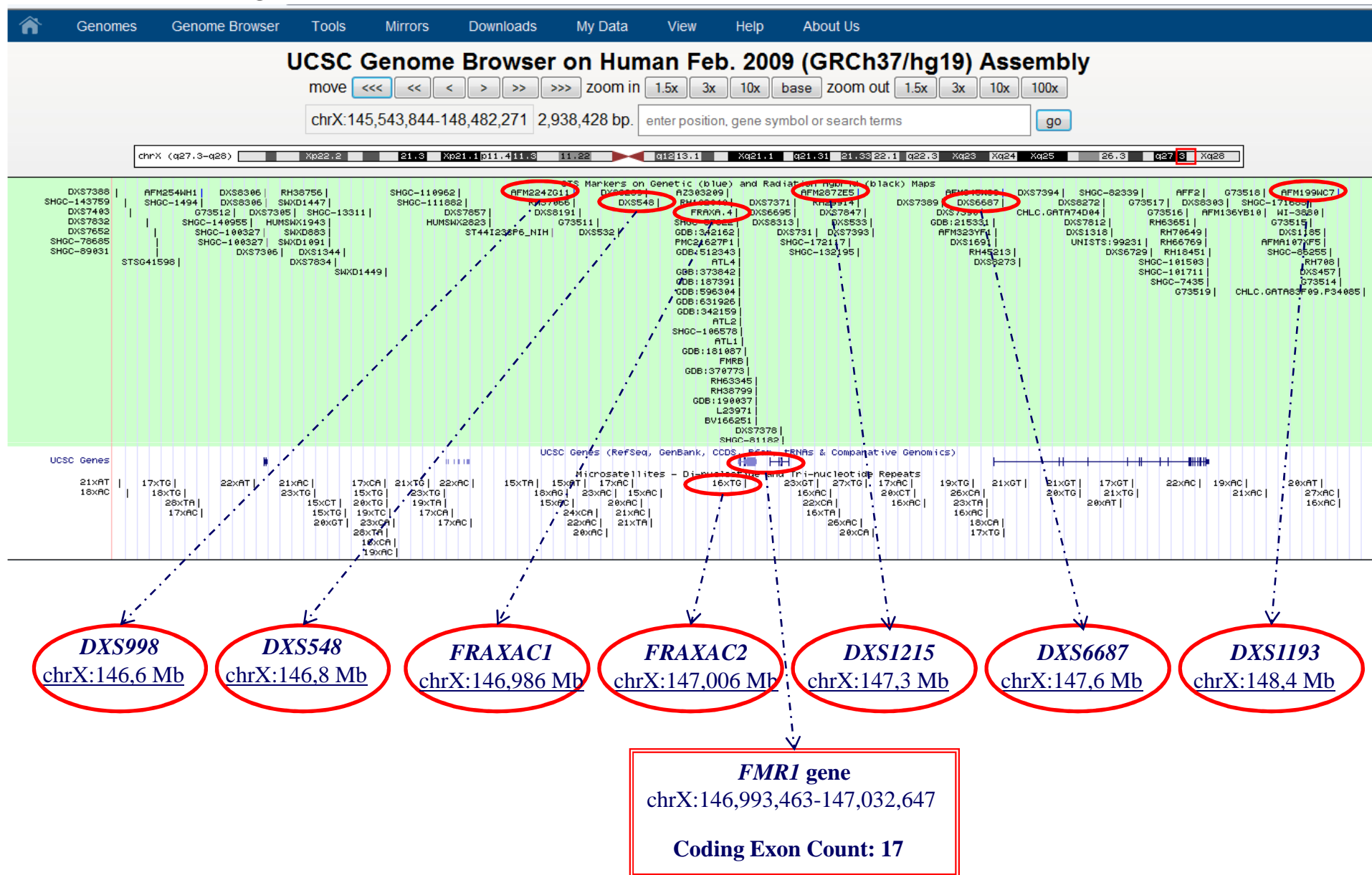

Supplement: Supplementary file 1 — Supplementary figures 1 and 2 show the specific location of the FMR1 gene and the STR markers used for PGD of FRAX. [file 965839.f1.pdf]
